# Synthesis, Characterization, and Biodistribution of Quantum Dot-Celecoxib Conjugate in Mouse Paw Edema Model

**DOI:** 10.1155/2018/3090517

**Published:** 2018-03-22

**Authors:** Suresh K. Kalangi, Nitin K. Swarnakar, R. Sathyavathi, D. Narayana Rao, Sanyog Jain, Pallu Reddanna

**Affiliations:** ^1^University of Hyderabad, Department of Animal Biology, School of Life Sciences, Hyderabad 500 046, India; ^2^National Institute of Pharmaceutical Education and Research (NIPER), Centre for Pharmaceutical Nanotechnology, Department of Pharmaceutics, Sector 67, S.A.S. Nagar, Mohali, Punjab 160062, India; ^3^University of Hyderabad, School of Physics, Hyderabad 500 046, India

## Abstract

Increased risk of cardiovascular side effects has been reported with many of the drugs in the market, including nonsteroidal anti-inflammatory drugs (NSAIDs). Hence, it is critical to thoroughly evaluate the biodistribution and pharmacokinetic properties of the drugs. Presently nanotechnology in combination with noninvasive imaging techniques such as magnetic resonance imaging (MRI), computed axial tomography (CAT), and positron emission tomography (PET) provides a better estimate of the spatio-temporal distribution of therapeutic molecules. Optical imaging using quantum dot- (QD-) tagged biological macromolecules is emerging as a fast, economical, sensitive, and safer alternative for theranostic purposes. In the present study, we report the nanoconjugates of mercaptopropionic acid- (MPA-) capped CdTe quantum dots (QDs) and Celecoxib for bio-imaging in carrageenan-induced mouse paw edema model of inflammation. QD-Celecoxib conjugates were characterized by fluorescence, FT-IR, NMR, and zeta-potential studies. *In vivo* imaging of QD-Celecoxib conjugates showed clear localization in the inflamed tissue of mouse paw within 3 h, with a gradual increase reaching a maximum and a later decline. This decrease of fluorescence in the paw region is followed by an increase in urinary bladder region, suggesting the possible excretion of QD-drug conjugates from mice within 24 h.

## 1. Introduction

NSAIDs are promising anti-inflammatory molecules in treating inflammatory disorders with the recent focus on cancer therapy [1]. In each year, above 40% of elderly people of 65 years have NSAIDs in their doctor's prescription in the USA alone [2, 3], because of NSAIDs' efficacy and common availability in treating the pain in age-associated diseases like osteoarthritis and other musculoskeletal disorders [3]. 41,000 hospitalizations and 33,000 deaths each year among older adults are reported to be due to the chronic usage of NSAIDS and their adverse reactions [4]. It is a well-established fact that chronic usage of most of NSAIDs is associated with increased risk of renal failure, peptic ulcers, myocardial infarction, stroke, and cerebrovascular and CNS-related adverse effects in older adults [3, 5–8]. Despite gastrointestinal toxic effects of NSAID usage [9, 10], withdrawal of Rofecoxib has brought the potential cardiac risks in the limelight [9, 11, 12], especially among the adults aged 65 years and above [6]. Celecoxib, a next-generation NSAID and the first selective COX-2 inhibitor, has been approved for use in the USA for the relief of signs and symptoms of rheumatoid arthritis and osteoporosis in adults [6]. In addition to its analgesic, antipyretic, and anti-inflammatory activities, recent studies have shown its chemopreventive and chemotherapeutic properties against a variety of cancers [1]. It is now believed that selective inhibition of COX-2, next-generation coxibs, even causes upper GI toxicity (lower than that of traditional NSAIDs), cardiac risks, and other problems [6, 11].

Currently available noninvasive imaging techniques for the evaluation of drug efficacy and safety, in preclinical research, either require the use of hazardous radiolabelled tracers or is expensive and time-consuming. Therefore, the development of economical, sensitive, and faster alternative approaches for the determination of spatio-temporal distribution of drug molecules is essential [13]. In the recent past, selective fluorescent inhibitors of COX-2 were developed alternatively for fluorine-radiolabelled agents in cancer diagnosis by PET imaging [14–16]. Conjugating drugs to fluorophores may alter drug property, and selective tagging of drugs to fluorophores is always a limitation as fluorophores have limited functional groups for conjugation. Hence, it is unavoidable to prepare drug molecules as imaging agents by attributing fluorescent properties every time, which is expensive and time-consuming [17]. Optical imaging in combination with nanotechnology provides an exciting opportunity in this direction. Quantum dots and nanoscale fluorophores conjugated to biological macromolecules have been successfully used in theranostic studies. However, their use in pharmacokinetic and biodistribution studies remains to be explored.

With increasing evidences of nanoparticles as powerful molecular imaging agents [18–20], the above limitations could be achieved by using the fluorescent semiconductor nanoparticles called quantum dots (QDs). QDs have distinctive optical properties such as advanced fluorescence lifetime, tunable size, increased photobleach thresholds, and capacity of multiplex imaging, with no match among the existing conventional dyes [20–27]. Developing a novel quantum dot-based molecular imaging method is one of the best choices to avoid the accumulation of nonspecific probe and can also increase the accuracy of diagnosis [20, 21, 28–32]. Unlike conventional fluorophores, the QDs, being inert fluorescent nanoparticles, form a unique kind of engineering platform with a wide range of capping molecules for bio-conjugation of drugs without altering the drug property [20, 21, 23, 26, 33]. With a wide range of selective tagging options, QDs not only work as a single tracer for multiple bioactive molecules but can also be utilized in multiple functions like specific target imaging, delivery, and tracing [20, 23, 32–35]. Though similar kinds of efforts were made with naproxen conjugated to QDs, QD-naproxen conjugates failed to enter the cells [36]. Later, targeting the alveolar macrophages in mice through doxorubicin-conjugated QDs has also been demonstrated [37]. Our previous study also demonstrated the tracing of the 5-fluorouracil-conjugated QDs in MCF-7 cancer cells [38].

In the present study, we explored the possibility to test the capability of QD-Celecoxib conjugate to be an alternative to radioisotopes in detecting the inflammation in mice paw edema model. It is also expected to map off targets and *in vivo* distribution of QD-Celecoxib conjugate through molecular imaging. To the best of our knowledge, the present study is the first of its kind with covalent tagging of NSAIDs with QDs.

## 2. Materials and Methods

### 2.1. Materials

Cadmium chloride hemi(pentahydrate), tellurium powder (99.99%), sodium borohydride, 3-mercaptopropionic acid (MPA), sodium hydroxide (NaOH), 1-ethyl-3-[3-dimethylaminopropyl]carbodiimide hydrochloride (EDC), and carrageenan were purchased from Sigma, USA. Celecoxib is a generous gift from Aurobindo Pharma Pvt Ltd., Hyderabad, India.

### 2.2. Methods

#### 2.2.1. Preparation of Water Soluble MPA-Capped CdTe QDs

CdTe QDs were prepared using the reaction between Cd_2_^+^ and NaHTe solution in the presence of 3-mercaptopropionic acid (MPA) as a stabilizer according to the published procedures, with little modifications [38, 39]. At the first stage, with a molar ratio of 2 : 1, sodium borohydride was used to react with tellurium in water to prepare sodium hydrogen telluride (NaHTe). At the second stage, the freshly prepared oxygen-free solution of NaHTe was added to nitrogen-saturated 1.25 mM CdCl_2_·2.5H2O and dissolved in 100 ml of water. To this solution, 3 mM of the MPA was added under stirring, followed by adjustment of the pH to 11.2 by dropwise addition of 1 M NaOH. The molar ratio of Cd_2_^+^:MPA:HTe^−^ was 1 : 2.4 : 0.5. The reaction mixture was placed in a three-necked flask fitted with a septum and valves and is deaerated by N_2_ bubbling for 45 min. Under stirring, NaHTe is passed through the solution together with a slow nitrogen flow for 20 min. CdTe precursors were formed at this stage under these conditions. The resulting mixture was refluxed at 110°C under open-air conditions with a condenser attached for 90 minutes to obtain CdTe nanocrystals of desired size.

#### 2.2.2. Conjugation of Celecoxib to QDs

QD-Celecoxib conjugate was prepared by reaction of MPA-capped CdTe QDs and Celecoxib in the presence of known cross-linker EDC (1-ethyl-3-[3-dimethylaminopropyl]carbodiimide hydrochloride) as base in phosphate buffer (pH 7.5) at room temperature with constant mixing for 2 h in a molar ratio of 1 : 1 : 0.5 (Celecoxib:EDC:QDs). Further, QD-Celecoxib conjugates (10 nM, 100 nM, 1 *μ*M, and 10 *μ*M of Celecoxib) were also prepared without changing the QD and EDC concentrations. Scheme 1 (refer to Supplementary Materials available here) shows the proposed hypothesis of bio-conjugation between NH_2_ groups of Celecoxib with carboxyl groups of MPA on CdTe through EDC [36, 40]. For all further studies, out of all the prepared conjugates, 100 *μ*M of Celecoxib conjugate (50 *μ*g/ml equivalent to QD weight) was used until it is specified. After the conjugation process, QD-Celecoxib conjugates were washed twice to remove unreacted molecules with PBS (pH 7.4) by centrifuging at 1500*g* for 20 min for further use.

#### 2.2.3. Fluorescence Spectroscopy

Emission measurements were recorded for QDs and different concentrations of QD-Celecoxib conjugates (*λ*_ex_ = 400 nm and *λ*_em_ **=** 529 nm) at room temperature with a Fluorolog-3 (HORIBA Jobin Yvon, FL3-221).

#### 2.2.4. FT-IR

The IR spectra were recorded on a JASCO FT-IR model 5300 for MPA-capped QDs (50 *μ*g/ml) and QD-Celecoxib (50 *μ*g/ml equivalent to QD weight) conjugates in PBS at pH 7.4.

#### 2.2.5. Solid-State NMR

^13^C solid-state NMR (100 MHz) spectra for MPA-capped QDs (50 *μ*g/ml) and QD-Celecoxib conjugates (50 *μ*g/ml) and Celecoxib were recorded on Bruker AVANCE 400 spectrometer. QDs and QD-Celecoxib conjugates were precipitated with ethanol, dried overnight at 50°C, and used for NMR studies.

#### 2.2.6. Size and Zeta-Potential Studies

Size distribution and zeta potential of the QDs and Celecoxib conjugates were measured by using Malvern Zetasizer v6.20 in PBS at pH 7.4.

#### 2.2.7. Biodistribution Studies of QD-Celecoxib Conjugates *In Vivo*

*(1) Mouse Paw Edema Model*. Biodistribution and *in vivo* localization studies of QD-drug conjugates were carried out in Swiss albino mice weighing 25–30 g. All the animal study protocols were duly approved by the institutional ethical committee. Food and water for animals were made available throughout the experiment. Edema was induced in mice by subcutaneous injection of carrageenan (0.1 ml of 1% w/v solution in normal saline) into the subplantar region of the left hind paw [41]. Edema-induced animals were randomly divided into 3 groups, each containing three mice.

Group I: This group of animals received PBS and served as the control group.
Group II: This group of animals received MPA-capped CdTe QDs at a dose of 2.5 mg/kg body weight.
Group III: This group of animals received (100 *μ*M) QD-Celecoxib conjugates at a dose of 2.5 mg (equivalent to QD weight)/kg body weight.

After appropriate treatments, the animals were observed for fluorescence under *in vivo* imaging at different time periods (0, 6, 12, and 24 h). All formulations were administered by intravenous route through tail vein injection of PBS at pH 7.4 [42].

*(2) In Vivo Fluorescence Imaging*. All mice were given anaesthesia by IP injection of 80 mg/kg body weight of ketamine (Ketajet Sterfil Laboratories Pvt. Ltd, India). Different groups of mice were imaged for the distribution of only QDs or Celecoxib QD conjugates using PhotonIMAGER (Biospace, France). *In vivo* fluorescence imaging was taken as explained by Swarnakar et al. [43].

## 3. Results

### 3.1. Characterization of QD-Drug Conjugates

**Figure 1:**
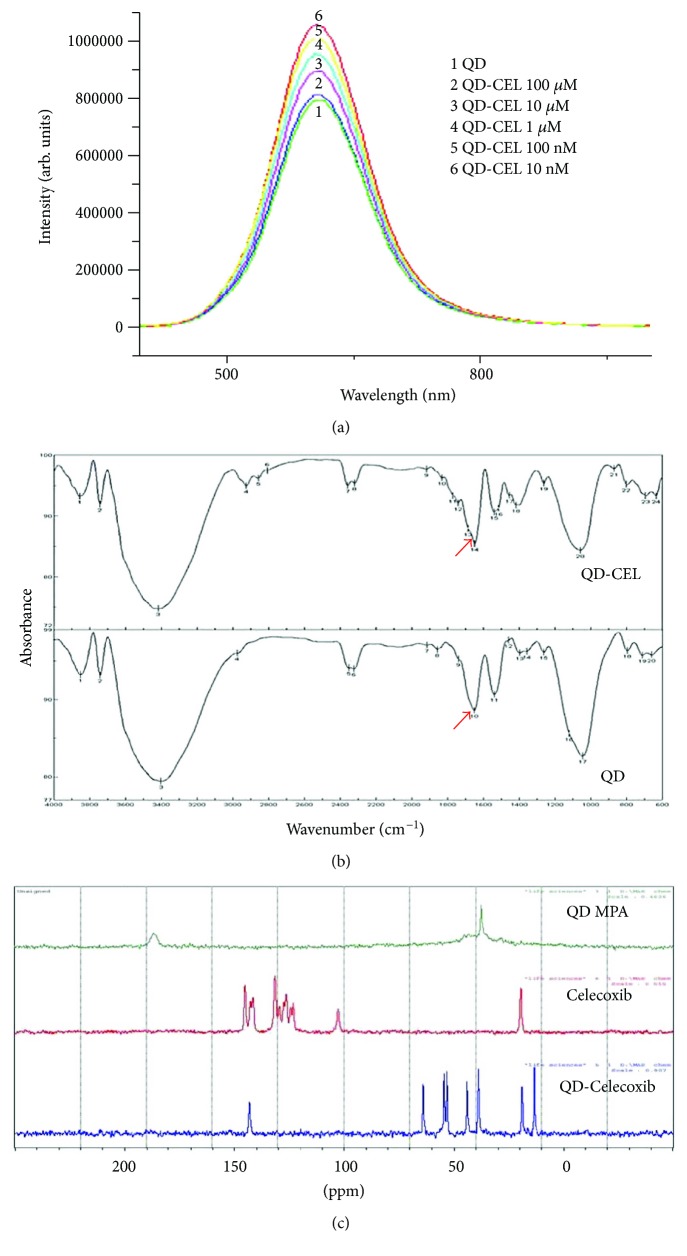
(a) Emission spectra of QD-Celecoxib conjugates (*λ*_ex_ **=** 400 nm and *λ*_em_ **=** 529 nm). Emission spectra showing concentration-dependent fluorescence increase (10 nM > 100 nM > 100 *μ*M > 10 *μ*M > 100 *μ*M) of Celecoxib-conjugated QDs (50 *μ*g/ml) over unconjugated QDs (50 *μ*g/ml). (b) FT-IR spectra of QD-Celecoxib conjugates with shifting of peak from 1640 cm^−1^ (C=O) to 1540 cm^−1^ showing the occupancy of Celecoxib of QD-drug conjugates against unconjugated MPA-capped QDs. (c) ^13^C solid-state NMR of Celecoxib, MPA-capped QDs, and QD-Celecoxib conjugate. Spectra of QD-Celecoxib conjugate (bottom) showing more number of carbons in comparison to Celecoxib (centre) and QDs alone (top), which forms an indirect confirmation of conjugation between Celecoxib and QDs.

The quality of the synthesized CdTe MPA QDs was assessed by measuring the average mean diameter and zeta potential of the nanoparticles. The mean diameter and zeta potential as measured by dynamic light scattering of nanoparticles, using Zetasizer, were found to be ~8 nm and −20.4 mV, respectively (refer to Supplementary Materials). The fluorescence characteristics of the synthesized QDs were confirmed by their fluorescence emission spectra (Figure 1(a)). Thus characterized QDs were used for tagging the Celecoxib, an anti-inflammatory drug. The conjugation of Celecoxib to QDs was done as described in Methods, and the conjugation was confirmed by FT-IR and NMR spectroscopies. The formation of QD-Celecoxib conjugates was confirmed by FT-IR spectroscopy. A significant shift in the peak from 1640 cm^−1^ (C=O) to 1540 cm^−1^ has been observed in the FT-IR spectrum, indicating the formation of the amide bond between the –COOH group of MPA on QDs and the –NH_2_ group of Celecoxib (Figure 1(b), top panel) [44]. This was further substantiated by the additional peaks corresponding to extra carbon atoms in ^13^C solid-state NMR spectra of the conjugates but not observed in unconjugated QDs (Figure 1(c)). Further, the formed conjugates have also shown a zeta potential of −28.5 mV, which is significantly different from that of unconjugated QDs (refer to Supplementary Materials). The observed change in the value of zeta potentials of MPA-capped QDs and QD-Celecoxib conjugates also confirms QDs and Celecoxib bio-conjugation [37]. However, no aggregation was observed in both samples, which indicates the good dispersion stability in PBS at pH 7.4. The effect of Celecoxib conjugation on the fluorescence properties of QDs was assessed by evaluating the fluorescence emission spectra of QD-Celecoxib conjugates synthesized by incubating different concentrations of Celecoxib with QDs. Although we have not observed any significant change in the excitation/emission wavelengths of the spectra, there was a significant decrease in the peak fluorescence intensity with an increase in the Celecoxib concentration (Figure 1(a)). The observed decrease may be attributed to the number of –COOH groups occupied by the Celecoxib molecules.

### 3.2. Evaluation and Biodistribution of QD-Celecoxib Conjugates *In Vivo*: Mouse Paw Edema Model

**Figure 2:**
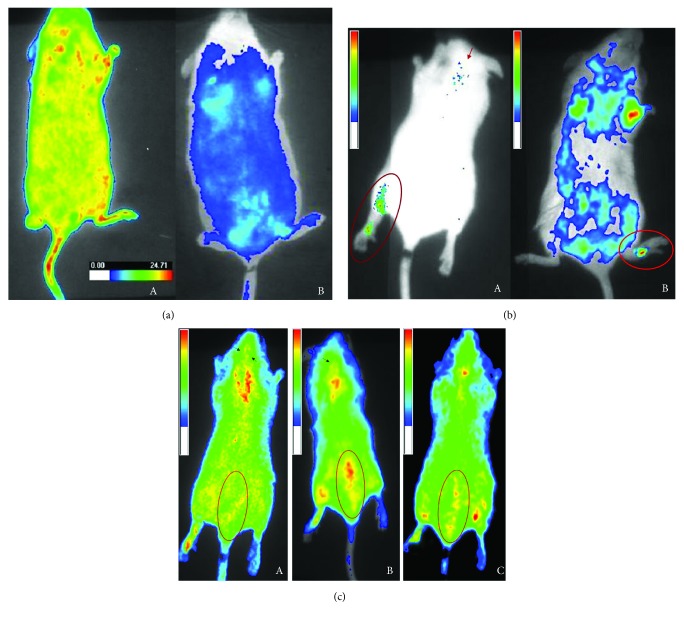
(a) *In vivo* imaging of unconjugated CdTe MPA QDs (2.5 mg/kg body wt.) in mouse paw edema model showing evenly distributed fluorescence in the whole body at 0 h (left); *in vivo* imaging of mouse shows no significant fluorescence specifically localized anywhere in the body at 24 h (right). (b) *In vivo* imaging of QD-Celecoxib conjugates (2.5 mg/kg body wt.) at 3 h of posttreatment in mouse paw edema model showing the clear localization of QD-Celecoxib conjugates in inflamed tissue of paw edema, and the arrow indicates may be some deposition in the brain region also in dorsal view (left); ventral view of same mouse showing localization of QD-Celecoxib conjugates near the heart region other than in the paw edema tissue (right). (c) *In vivo* imaging of mice treated with QD-Celecoxib conjugates (2.5 mg/kg body wt.) at different time intervals: (A) 6 hrs, (B) 12 h, and (C) 24 h postinjection of QD-Celecoxib conjugates through tail vein. An increase in fluorescence intensity near the urinary bladder (circled area) with increasing time up to 12 h and a later decrease at 24 h can be observed that. Arrows in (A) and (B) are thought to be the deposition of QD-Celecoxib conjugates in the brain region, which cleared after 12 h.

In order to evaluate the efficiency of drug-conjugated QDs, as potential alternative to radioactive drug tracers, in pharmacokinetic studies, we introduced, by an intravenous injection, unconjugated/Celecoxib-conjugated QDs into mice with left hind paw edema. The mice receiving an intravenous injection of PBS were maintained as controls. *In vivo* imaging of mice with inflamed paw injected with unconjugated QDs shows the uniform distribution of fluorescence (Figure 2(a)) all over the mice body with no significant localization to inflamed paw or any other part of the body. The mice group which received the QD-Celecoxib conjugates showed significant localization of QD-Celecoxib conjugates in paw edema tissue (Figure 2(b)). This demonstrates the clear targeting of the QD-Celecoxib conjugates to the inflammatory tissue of mice. However, the intensity of fluorescence was greatly decreased all over the mice except the inflamed paw with increase in the duration of exposure, 6 h, 12 h, and 24 h. On the other hand, florescence was increased in the lower abdominal region, near the urinary bladder, suggesting clearance of QD-Celecoxib conjugates from mouse body as shown in Figure 2(c).

## 4. Discussion

*In vivo* imaging of drugs forms a powerful approach to monitor the metabolism and excretion of drug molecules. Monitoring the behaviour of a single molecule in living cells is a powerful approach to investigate the details of cellular processes [35, 45]. Radioisotope tracers have been widely used in modern pharmacology for pharmacokinetic and pharmacodynamic studies in experimental animal models. Though radioisotopes are the only suitable option for studying biodistribution and pharmacokinetics of drugs at a single-molecule level, there are several drawbacks with these tracers, warranting the development of more sensitive tracers for multiplexed imaging, with high photobleaching threshold and quantitative determination during biomedical research applications. In connection to this, the quantum dot- (QD-) drug conjugates have shown promise in applications spanning both diagnostics and therapeutics [36]. Here, we report a QD-based approach by molecular imaging for drug molecule tracing and biodistributional studies in mice. Studies were taken up to evaluate the selectivity of QD-anti-inflammatory drug conjugates in mouse paw edema model.

Celecoxib, a selective COX-2 inhibitor, is being used extensively for inflammatory disorders because of its low gastrointestinal side effects as compared to the conventional NSAIDs, despite its known cardiac side effects [46]. We have successfully developed a method for conjugation of Celecoxib to QDs and characterization. As expected, QD-Celecoxib conjugates were mainly localized in paw edema of mice. This indicates that Celecoxib retained its drug property to lead the QDs to localize at the inflammatory tissue, where COX-2 is known to be overexpressed. However, no such localization was observed in case of unconjugated MPA-capped QDs as shown in 24 h *in vivo* image. From these studies, it is clear that QD-Celecoxib conjugates are capable of tracing the inflammatory tissue in mice as shown by molecular imaging. In addition to the localization of QD-Celecoxib conjugates at the inflamed tissue, fluorescence was also observed near the heart and brain tissue of mice treated with QD-Celecoxib conjugates. This may explain the reported cardiac side effects of Celecoxib and other coxibs [12, 46, 47]. A number of studies have shown the constitutive expression of COX-2 in the brain [48, 49], which may explain the observed localization of QD-Celecoxib conjugates in the brain tissue also. QDs, being very small in size (2–10 nm), are capable of crossing the blood-brain barrier [50]. In view of the fast clearance of QDs over 24 h from the body, it makes them less toxic than radioisotopes, and their renal excretion of QDs was well explained by Choi et al. in mice [51]. As existing reports say that adverse reactions of NSAIDs among the elderly people are due to the age-related loss of organ reserve, high comorbidities, polypharmacy, and altered pharmacokinetics [52], we believe that platforms like this can increase the chance of understanding the tracing, biodistribution, and drug reach to the target in small animal studies at laboratory-level drug screening.

## 5. Conclusion

This study presents data on conjugation of CdTe QDs with Celecoxib, a selective COX-2 inhibitor and their successful application in bio-imaging and whole body distribution studies in paw edema models of inflammation in mice... Interestingly this approach has revealed the off targets of Celecoxib which may explain its reported side effects. The method employed in the present study for Celecoxib could be employed for other drug candidates to find their target and nontarget interactions in animal models. This approach stands unique, as it not only helps in bio-imaging for drug distributional studies and also to map possible off targets.

## Supplementary Materials

Supplementary Materials Scheme 1: celecoxib conjugation with the MPA-capped CdTe QDs. MPA-capped CdTe QDs (1 mg/ml in PBS at pH 7.4) size distribution by Zetasizer showing a major peak at ~8 nm with high intensity and also a small pool of particles showing above 100 nm. Zeta-potential studies for unconjugated and Celecoxib-conjugated QDs showing −20.4 mV for QD-Celecoxib conjugates and −28.5 mV for MPA-capped QDs.
