# Supplementary material for: Synthesis, Characterization, and Biodistribution of Quantum Dot-Celecoxib Conjugate in Mouse Paw Edema Model
Source: Oxid Med Cell Longev. 2018 Mar 22;2018:3090517. doi: 10.1155/2018/3090517 (PMC6038454; doi:10.1155/2018/3090517)
Supplement: Supplementary Materials — Scheme 1: celecoxib conjugation with the MPA-capped CdTe QDs. MPA-capped CdTe QDs (1 mg/ml in PBS at pH 7.4) size distribution by Zetasizer showing a major peak at ~8 nm with high intensity and also a small pool of particles showing above 100 nm. Zeta-potential studies for unconjugated and Celecoxib-conjugated QDs showing −20.4 mV for QD-Celecoxib conjugates and −28.5 mV for MPA-capped QDs. [file 3090517.f1.pdf]

Supplementary Material:

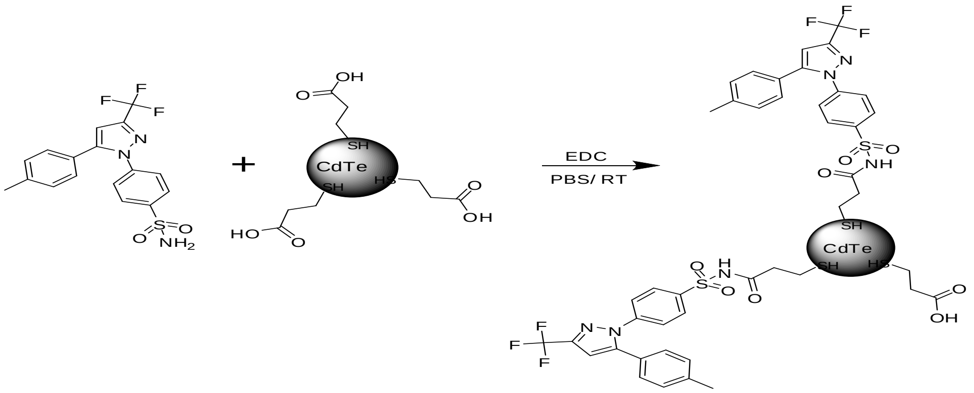

Celecoxib **MPA capped QDs** **QD-Celecoxib conjugates**

Scheem.1. Celecoxib conjugation with the MPA capped CdTe QDs

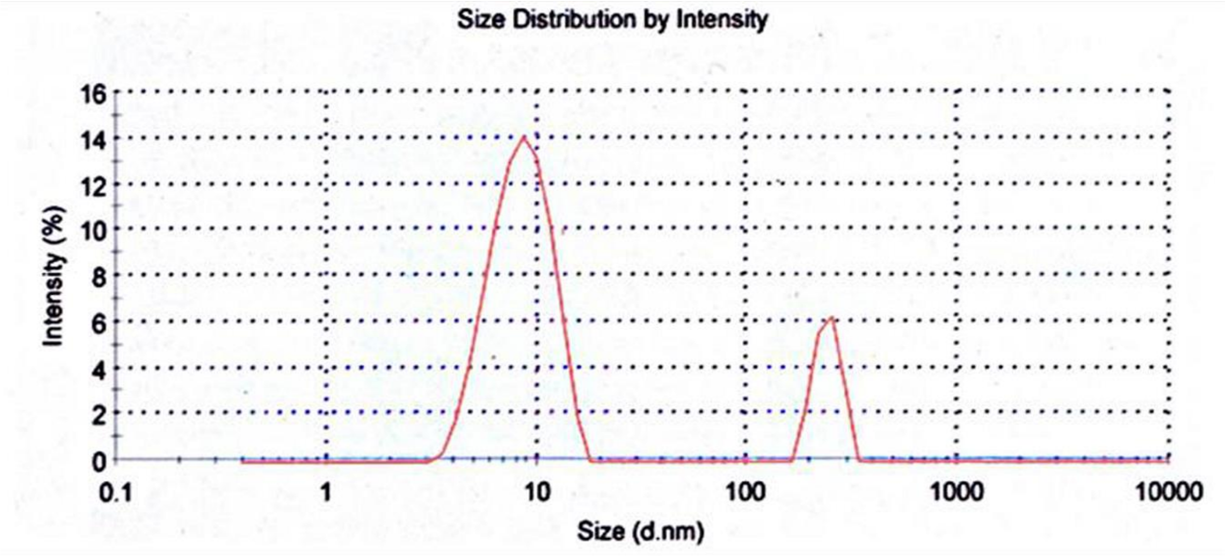

MPA capped CdTe QDs (1mg/ml in PBS at pH 7.4) size distribution by Zetasizer showing major peak at ~8 nm with high intensity and also a small pool of particles showing above 100nm.

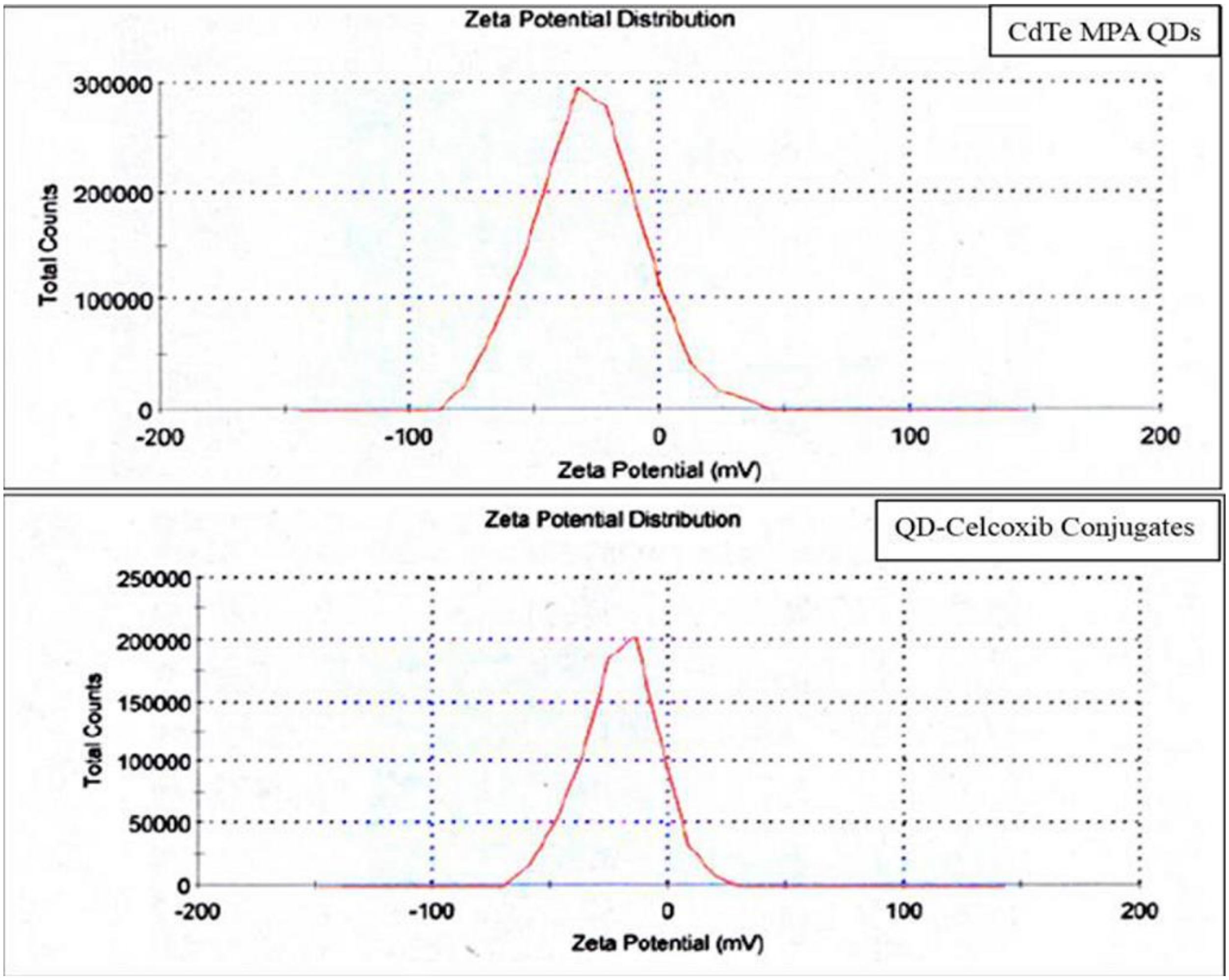

Zeta potential studies for un-conjugated and Celecoxib conjugated QDs showing - 20.4 mV for QD-Celelcoxib conjugates - 28.5 mV for MPA capped QDs
